# Integrative functional bioinformatic analysis of NAD^+^-associated protein coding genes in adipose tissue (dys)function – evidence from targeted rodent transcriptomic studies

**DOI:** 10.3389/fendo.2026.1807667

**Published:** 2026-07-21

**Authors:** Filip Postolov, Dragan Milenkovic, Tatjana Ruskovska

**Affiliations:** 1 Faculty of Medical Sciences, Goce Delcev University, Stip, North Macedonia; 2 Plants for Human Health Institute, Department of Food Bioprocessing & Nutrition Sciences, North Carolina State University, Kannapolis, NC, United States

**Keywords:** adipose tissue dysfunction, bioinformatics, differentially expressed genes, insulin resistance, NAD, pathway enrichment analysis, protein-protein interactions, transcriptomics

## Abstract

One of the key regulatory mechanisms in adipose biology is the NAD^+^/SIRT axis, which is disrupted in conditions such as obesity and insulin resistance. In recent years, increasing attention has been directed toward understanding the role of NAD^+^ in adipose biology, including through gene expression analyses. However, most published studies have focused on a limited set of preselected genes, and an integrated analysis of genes associated with NAD^+^ alterations in adipose tissue is lacking. The aim of this study was to conduct a systematic literature search to identify rodent studies reporting significant modulation of NAD^+^ levels in adipose tissue, along with significant changes in gene expression within the same adipose depot. The extracted genes were then subjected to integrative functional bioinformatic analyses to identify significantly enriched canonical pathways, protein-protein interactions, master regulators, and associations with human diseases. We identified 17 studies, from which 113 unique NAD^+^-associated protein coding genes were extracted. Pathway enrichment analysis revealed multiple canonical pathways significantly associated with these genes, including *thermogenesis*, the *PPAR signaling pathway*, the *AMPK signaling pathway*, and *adipogenesis*. In addition, several cellular pathways were identified for which direct experimental evidence of NAD^+^ involvement is currently lacking, such as the *apelin signaling pathway* and the *relaxin signaling pathway*. Protein-protein interaction analysis revealed three distinct protein clusters related to *positive regulation of cold-induced thermogenesis*, *inflammation*, and *cellular respiration*. Master regulator analysis identified several well-established regulators, such as leptin, PRDM16, PPARGC1A, AMP, glucose, fructose, and rosiglitazone, as well as regulators that have not yet been directly investigated in the context of NAD^+^ function in adipose biology. Finally, analysis of disease associations of NAD^+^-associated protein coding genes revealed links to nutritional and cardiometabolic diseases. In conclusion, this study highlights both well-established and previously unexplored canonical pathways and regulators of biological processes associated with the role of NAD^+^ in adipose biology, thereby identifying potential targets for future experimental investigation. Additionally, our systematic literature search revealed a notable lack of studies employing global transcriptomic approaches, as well as multi-omics studies with coupled bioinformatic analyses, which are essential for obtaining an in-depth and comprehensive understanding of the role of NAD^+^ in adipose biology.

## 1 Introduction

Adipose tissue, once regarded solely as a passive lipid-storage compartment, is now recognized as a dynamic endocrine organ that plays a fundamental role in the regulation of whole-body energy homeostasis in response to diverse metabolic and environmental stimuli (1). Functional, non-obese adipose tissue is insulin-sensitive and promotes whole-body insulin sensitivity. In obesity, however, adipose tissue undergoes dramatic phenotypic, metabolic, and histological alterations that lead to inflammation and dysfunction, contributing to systemic metabolic dysregulation and increasing the risk of type 2 diabetes and associated cardiometabolic disorders (2, 3). Accumulating evidence further indicates that adipose tissue acts as an early responder to aging and plays a significant role in driving systemic aging processes (4). These functions are largely mediated by the secretory activity of adipose tissue, which releases a broad spectrum of bioactive molecules collectively referred to as adipokines (5). In addition to adipokines, the adipose tissue secretome includes lipokines, extracellular vesicles and microRNAs (miRNAs), which exert important regulatory effects on key biological processes in both health and disease (6, 7).

Adipose tissue exhibits a highly complex structure and cellular composition. In addition to mature adipocytes, which make up more than 90% of adipose tissue volume but represent less than 50% of its cellular content, adipose tissue is rich in adipocyte progenitor cells and multiple populations of immune cells (8). Based on adipocyte morphology and metabolic function, mammalian adipose tissue is broadly categorized into three distinct phenotypes: white adipose tissue (WAT), brown adipose tissue (BAT), and beige adipose tissue (bAT) (9). The primary physiological role of WAT is the storage of metabolic energy as triglycerides. Accordingly, white adipocytes contain large unilocular lipid droplets and exhibit low mitochondrial density. In addition to energy storage, WAT functions as an endocrine organ by secreting adipokines, including leptin, adiponectin, and resistin, that regulate appetite, insulin sensitivity, inflammation, and systemic metabolic homeostasis (10). In contrast, BAT is specialized for thermogenesis, contains adipocytes with small multilocular lipid droplets, and exhibits a high mitochondrial density. Its hallmark, non-shivering thermogenesis, is driven by uncoupling protein 1 (UCP1), which resides in the inner mitochondrial membrane of brown adipocytes and mediates dissipation of electrochemical gradient as heat rather than allowing it to fuel ATP synthesis (9, 10). Beige adipose tissue is a “hybrid” form that shares features of both WAT and BAT. Beige adipocytes arise either through the recruitment of a specific preadipocyte subpopulation within WAT or *via* the reversible brown remodeling, *i.e.*, browning, of white adipocytes in response to various physiological or pharmacological stimuli. Like brown adipocytes, beige adipocytes contain small multilocular lipid droplets and exhibit high mitochondrial density. They express UCP1, while also employing UCP1-independent mechanisms to convert chemical energy into heat (9, 10).

NAD^+^ (the oxidized form of nicotinamide adenine dinucleotide) has long been recognized as one of the central coenzymes in redox reactions, transferring reducing equivalents within major metabolic pathways (11, 12). Later studies, however, have revealed roles that extend far beyond its classical functions in redox chemistry (13). Notably, NAD^+^ serves as an essential co-substrate for sirtuins (SIRT), a highly conserved class of NAD^+^-dependent deacetylases that also function as lysine deacylases and mono-ADP-ribosyltransferases (14). Multiple studies have demonstrated that the interplay between NAD^+^ and sirtuins constitute an essential regulatory system that modulates inflammation, metabolism, oxidative stress, and apoptosis (15). Other non-redox NAD^+^-consuming reactions are catalyzed by CD38 and poly(ADP-ribose) polymerases (PARPs). CD38 is a transmembrane protein and multifunctional enzyme that converts NAD^+^ to cyclic ADP-ribose or ADP-ribose, thereby reducing tissue NAD^+^ levels (16). PARPs comprise a family of enzymes that catalyze mono- and poly-ADP-ribosylation, post-translational modifications in which ADP-ribose is transferred to target proteins. In these reactions, NAD^+^ serves as the source of ADP-ribose, through its cleavage into nicotinamide (NAM) and ADP-ribose (17). Collectively, participation in these non-redox reactions establishes NAD^+^ as one of the central regulators of cellular physiology.

In the adipose organ, as in other metabolically active tissues, maintaining sufficient intracellular NAD^+^ levels is essential for sirtuin activity, enabling the regulation of cellular homeostasis and energy metabolism. In inflamed obese adipose tissue, however, the NAD^+^/SIRT axis is significantly impaired potentially due to the upregulation of CD38 and PARPs, leading to increased NAD^+^ consumption and depletion of intracellular NAD^+^ pools (18). Importantly, downregulation of the NAD^+^/SIRT pathway at the gene expression level has been reported in subcutaneous adipose tissue in acquired obesity in humans and was negatively correlated with multiple measures of metabolic health, even before the development of overt metabolic disease (19). This perturbation of metabolic homeostasis predisposes to insulin resistance, type 2 diabetes mellitus, and a concomitant increase in cardiovascular risk (20).

Recent systematic literature search demonstrated that an increasing number of studies have explored the molecular mechanisms of NAD^+^ involvement in adipose tissue (dys)function (18). However, the majority of studies have relied on targeted gene expression analyses, thereby limiting investigations to a small, pre-selected set of genes defined by individual studies. Consequently, an integrated analysis of these fragmented data is lacking, and a comprehensive overview of the molecular signature associated with NAD^+^ modulation in adipose tissue remains absent. To address this gap, the aim of this integrating review was to expand our previous systematic literature search and identify studies reporting both significant modulation of NAD^+^ levels and concomitant changes in gene expression within the same adipose depot. After extracting NAD^+^-associated genes, we conducted an integrative functional analysis using multiple bioinformatic methods. This approach provides comprehensive mechanistic insight into the cellular pathways through which NAD^+^ contributes to adipose tissue physiology and pathophysiology. In the present work, we included only rodent models, given their relative abundance in the literature (18) and their suitability for elucidating the biological relevance of NAD^+^-related processes within the context of the whole organism.

## 2 Materials and methods

### 2.1 Literature search and data extraction

To address the objectives of our study, we conducted a systematic search of all available literature in the PubMed database using the keywords “nad” AND “adipo*”, from inception to October 3, 2024. Articles were included if they met the following criteria: (a) original research conducted in rodent models, (b) demonstration of significant modulation of NAD^+^ levels in adipose tissue accompanied by significant modulation of gene expression within the same adipose depot; and (c) use of a targeted analytical approach for gene expression analysis. Only articles published in English were included in our study.

Following the selection of eligible papers, differentially expressed genes were extracted into a template prepared specifically for this study. Their official gene names and symbols were identified using the Gene database, provided by the National Center for Biotechnology Information (NCBI) (https://www.ncbi.nlm.nih.gov/gene).

### 2.2 Functional bioinformatic analyses

Pathway enrichment analysis was performed using the bioinformatic tool GeneTrail3.2, which was employed as a platform for analyzing our gene set against *KEGG* and *WikiPathways* databases (21), accessed on April 17, 2025. The following settings were applied: over-representation analysis; null hypothesis for p-value computation: two-sided; method for p-value adjustment: Benjamini-Hochberg; significance level: 0.05; handling of potential duplicate entries in the uploaded file: median; reference: all supported genes. To corroborate the results obtained with GeneTrail, an additional pathway enrichment analysis was conducted using the bioinformatic tool Enrichr, analyzing the same gene set with the *WikiPathways 2024 Mouse* database (https://maayanlab.cloud/Enrichr; accessed December 8, 2025) (22).

Networks of gene-pathway interactions were built using the Cytoscape software, version 3.10.4 (23) (https://cytoscape.org; accessed December 12, 2025).

Protein-protein interaction (PPI) analysis was performed using the STRING database, version 12 (24) (https://string-db.org; accessed May 8, 2025). The following general settings were applied: network type: full STRING network; meaning of network edges: confidence (line thickness indicates the strength of data support); active interaction sources: textmining, experiments, databases, and co-expression; minimum required interaction score: high confidence (0.700); network display mode: static png. Subsequently, proteins were grouped into three distinct clusters using the k-means clustering functionality within STRING.

Master regulator analysis was performed by interrogating the Qiagen Ingenuity Pathway Analysis (IPA) database (Qiagen (https://digitalinsights.qiagen.com; accessed June 7, 2025). The analysis was conducted using the standard settings recommended by the manufacturer.

Comparative Toxicogenomics Database was interrogated to explore the association of extracted protein coding genes with human diseases (https://ctdbase.org; accessed May 6, 2025) (25).

## 3 Results

### 3.1 Selection of eligible studies and extraction of differentially expressed genes

**Figure 1:**
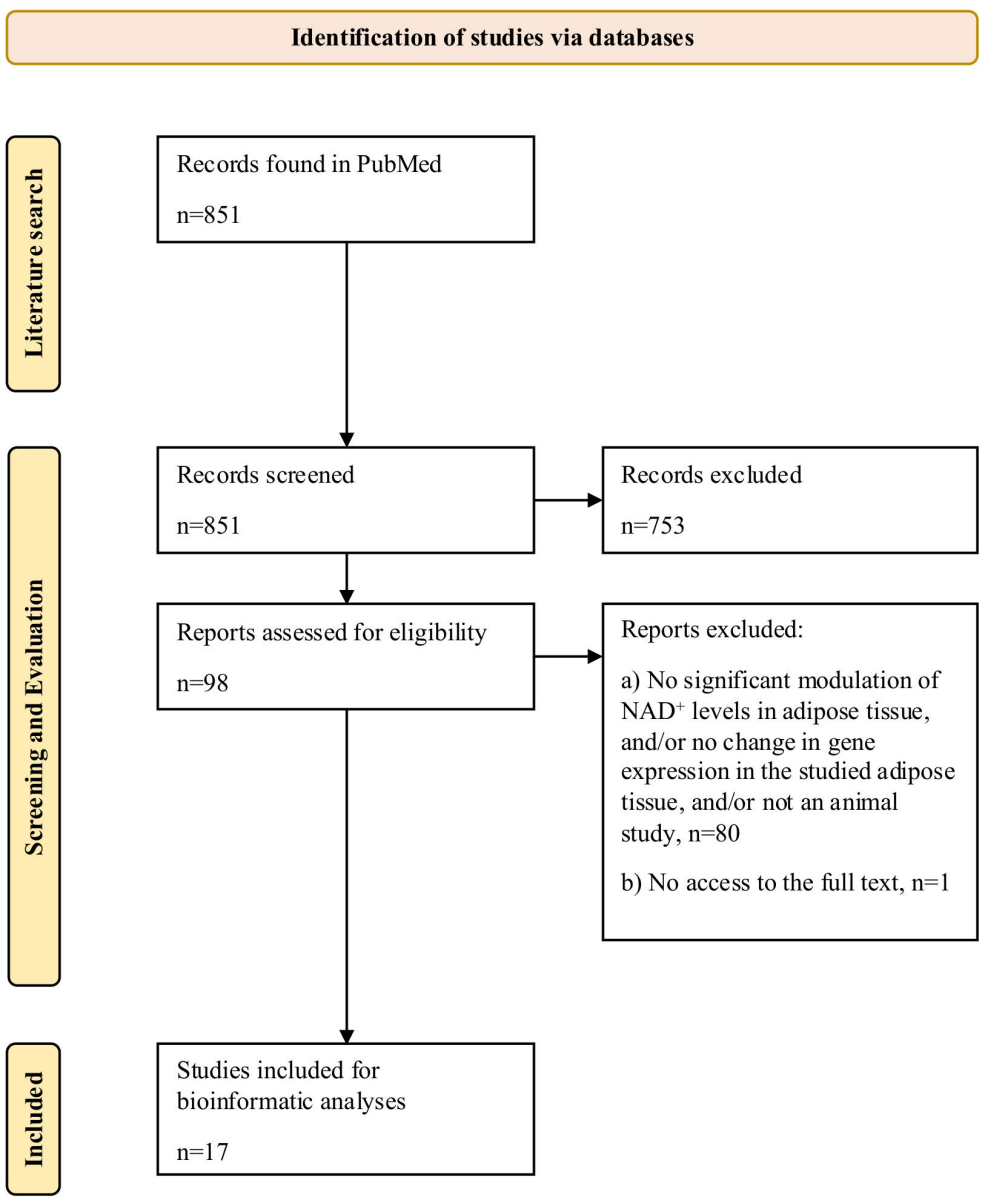
PRISMA diagram. Workflow of the selection process for eligible studies.

To identify eligible studies, a systematic search was conducted in the PubMed database using the predefined keywords. The search yielded a total of 851 records, which were screened based on the title and abstract according to the inclusion criteria outlined in the section *Materials and methods*. During the screening process, 753 records were excluded, and the remaining 98 were subjected to full-text review. Of these, 81 reports were excluded for the following reasons: (a) no significant modulation of NAD^+^ levels in adipose tissue and/or no change in gene expression in the analyzed adipose tissue and/or not being an animal study (n = 80); and (b) lack of access to the full text (n = 1). Consequently, 17 studies were included for data extraction and subsequent bioinformatic analyses. The PRISMA flow diagram (26), illustrating the systematic literature search and selection process is shown in **Figure 1**. A comprehensive overview of all included studies, as well as a detailed list of all extracted genes with their official symbols and names, is presented in **Table 1**.

**Table 1:** Overview of studies selected for functional bioinformatic analyses and detailed list of extracted genes with their official symbols and names.

No	Paper identification (DOI, title, and reference)	Species and model	Type of adipose tissue	Effects on NAD^+^ concentration in the relevant adipose tissue	NAD^+^-associated genes	
					Official gene symbol	Official gene name
1	DOI: 10.1093/gerona/glt122 Nicotinamide phosphoribosyltransferase is required for the calorie restriction–mediated improvements in oxidative stress, mitochondrial biogenesis, and metabolic adaptation (56)	Rat Calorie restriction (CR)	Visceral adipose tissue (VAT)	Increased NAD^+^ in VAT	*Nampt* *Nrf1* *Cox4i1* *Ppargc1a* *Tfam*	nicotinamide phosphoribosyltransferase nuclear respiratory factor 1 cytochrome c oxidase subunit 4i1 PPARG coactivator 1 alpha transcription factor A, mitochondrial
2	DOI: 10.1038/nature13198 Nicotinamide N-methyltransferase knockdown protects against diet-induced obesity (57)	Mouse Nnmt knockdown in high-fat diet mice	White adipose tissue (WAT)	Increased NAD^+^ in adipose tissue	*Amd1* *Odc1* *Sat1* *Fasn* *Acaca* *Nampt* *Nmnat2* *Nmnat3* *Cd36* *Cat* *Sdhb* *Gadd45a*	S-adenosylmethionine decarboxylase 1 ornithine decarboxylase, structural 1 spermidine/spermine N1-acetyl transferase 1 fatty acid synthase acetyl-Coenzyme A carboxylase alpha nicotinamide phosphoribosyltransferase nicotinamide nucleotide adenylyltransferase 2 nicotinamide nucleotide adenylyltransferase 3 CD36 molecule catalase succinate dehydrogenase complex, subunit B, iron sulfur (Ip) growth arrest and DNA-damage-inducible 45 alpha
3	DOI: 10.1016/j.jnutbio.2020.108377 Depot-specific regulation of NAD^+^/SIRTs metabolism identified in adipose tissue of mice in response to high-fat diet feeding or calorie restriction (58)	Mouse High-fat diet (Calorie restriction was also analyzed in this study. For clarity, we focused only on high-fat diet.)	Epididymal WAT Inguinal WAT Interscapular brown adipose tissue (iBAT) (Extracted are genes that have significant modulation in any of the studied fat depots.)	Decreased NAD^+^ in all studied fat depots	*Nampt* *Nmnat1* *Nmrk1* *Nnmt* *Aox1* *Cyp2e1* *Sirt1* *Sirt2* *Sirt3* *Sirt6* *Parp1* *Brinp1* *Ucp1* *Cidea* *Cox7a1* *Elovl3*	nicotinamide phosphoribosyltransferase nicotinamide nucleotide adenylyltransferase 1 nicotinamide riboside kinase 1 nicotinamide N-methyltransferase aldehyde oxidase 1 cytochrome P450, family 2, subfamily e, polypeptide 1 sirtuin 1 sirtuin 2 sirtuin 3 sirtuin 6 poly (ADP-ribose) polymerase family, member 1 bone morphogenic protein/retinoic acid inducible neural specific 1 uncoupling protein 1 (mitochondrial, proton carrier) cell death-inducing DNA fragmentation factor, alpha subunit-like effector A cytochrome c oxidase subunit 7A1 ELOVL fatty acid elongase 3
4	DOI: 10.1016/j.celrep.2016.07.027 NAMPT-mediated NAD^+^ biosynthesis in adipocytes regulates adipose tissue function and multi-organ insulin sensitivity in mice (43)	Mouse Adipocyte-specific Nampt knockout (ANKO)	Visceral adipose tissue (VAT) Subcutaneous adipose tissue (SAT)	Animal model; decreased NAD^+^ in WAT (59)	*Tnf* *Il6* *Ccl2* *Adgre1* *Cd68* *Adipoq* *Cfd* *Cidec* *Selenbp1* *Car3* *Cyp2f2* *Txnip* *Nr3c1* *Aplp2* *Nr1d1*	tumor necrosis factor interleukin 6 chemokine (C-C motif) ligand 2 adhesion G protein-coupled receptor E1 CD68 antigen adiponectin, C1Q and collagen domain containing complement factor D (adipsin) cell death-inducing DFFA-like effector c selenium binding protein 1 carbonic anhydrase 3 cytochrome P450, family 2, subfamily f, polypeptide 2 thioredoxin interacting protein nuclear receptor subfamily 3, group C, member 1 amyloid beta precursor-like protein 2 nuclear receptor subfamily 1, group D, member 1
5	DOI: 10.1073/pnas.1909917116 Adipose tissue NAD^+^ biosynthesis is required for regulating adaptive thermogenesis and whole-body energy homeostasis in mice (60)	Mouse Adipocyte-specific Nampt knockout (ANKO)	Brown adipose tissue (BAT) White adipose tissue (WAT)	Decreased NAD^+^ in BAT Animal model; decreased NAD^+^ in WAT (59)	*Sgk2* *Cyp2b10* *Ppargc1a* *Ucp1* *Dio2* *Ppara* *Lpl* *Cpt1b* *Gk* *Gpam* *Elovl6* *Scd1* *mt-Co1* *mt-Nd5* *Cav1* *Adrb3*	serum/glucocorticoid regulated kinase 2 cytochrome P450, family 2, subfamily b, polypeptide 10 peroxisome proliferative activated receptor, gamma, coactivator 1 alpha uncoupling protein 1 (mitochondrial, proton carrier) deiodinase, iodothyronine, type II peroxisome proliferator activated receptor alpha lipoprotein lipase carnitine palmitoyltransferase 1b, muscle glycerol kinase glycerol-3-phosphate acyltransferase, mitochondrial ELOVL fatty acid elongase 6 stearoyl-Coenzyme A desaturase 1 cytochrome c oxidase I, mitochondrial NADH dehydrogenase 5, mitochondrial caveolin 1, caveolae protein adrenergic receptor, beta 3
6	DOI: 10.1016/j.molcel.2019.12.002 Aifm2, a NADH oxidase, supports robust glycolysis and is required for cold- and diet-induced thermogenesis (61)	Mouse Cold exposure	Brown adipose tissue (BAT)	Animal model; increased NAD^+^ in iBAT (53)	*Aifm2* *Ucp1*	apoptosis-inducing factor, mitochondrion-associated 2 uncoupling protein 1 (mitochondrial, proton carrier)
		Mouse Global Aifm2-knockout	Brown adipose tissue (BAT) Inguinal WAT (iWAT)	Decreased BAT and iWAT NAD^+^/NADH ratio	*Ucp1* *Dio2* *Sox9* *Cebpb* *Cebpd* *Pparg*	uncoupling protein 1 (mitochondrial, proton carrier) deiodinase, iodothyronine, type II SRY (sex determining region Y)-box 9 CCAAT/enhancer binding protein beta CCAAT/enhancer binding protein delta peroxisome proliferator activated receptor gamma
		Mouse Aifm2-overexpression in UCP1^+^ cells	Brown adipose tissue (BAT) Inguinal WAT (iWAT)	Increased BAT and iWAT NAD^+^/NADH ratio	*Ucp1* *Dio2* *Cidea* *Sox9*	uncoupling protein 1 (mitochondrial, proton carrier) deiodinase, iodothyronine, type II cell death-inducing DNA fragmentation factor, alpha subunit-like effector A SRY (sex determining region Y)-box 9
7	DOI: 10.2337/db13-0518 PPARα and Sirt1 mediate erythropoietin action in increasing metabolic activity and browning of white adipocytes to protect against obesity and metabolic disorders (27)	Mouse Adipocyte-specific erythropoietin receptor (EpoR) knockout	Subcutaneous WAT (SWAT)	Decreased NAD^+^ in SWAT	*Cycs* *Idh3a* *Cpt1b* *Ppargc1a* *Cox7a1* *Epor^#^*	cytochrome c, somatic isocitrate dehydrogenase 3 (NAD^+^) alpha carnitine palmitoyltransferase 1b, muscle peroxisome proliferative activated receptor, gamma, coactivator 1 alpha cytochrome c oxidase subunit 7A1 erythropoietin receptor
		Mouse Erythropoietin treatment	Subcutaneous WAT (SWAT) and SWAT primary adipocytes	Increased NAD^+^ in SWAT	*Cycs* *Idh3a* *Cpt1b* *Ppargc1a* *Cox7a1* *Cidea* *Prdm16* *Ucp1* *Ucp3* *Ppara* *Retn* *Wfdc21* *Agt*	cytochrome c, somatic isocitrate dehydrogenase 3 (NAD^+^) alpha carnitine palmitoyltransferase 1b, muscle peroxisome proliferative activated receptor, gamma, coactivator 1 alpha cytochrome c oxidase subunit 7A1 cell death-inducing DNA fragmentation factor, alpha subunit-like effector A PR domain containing 16 uncoupling protein 1 (mitochondrial, proton carrier) uncoupling protein 3 (mitochondrial, proton carrier) peroxisome proliferator activated receptor alpha resistin WAP four-disulfide core domain 21 angiotensinogen
8	DOI: 10.1016/j.cmet.2011.03.004 PARP-1 inhibition increases mitochondrial metabolism through SIRT1 activation (62)	Mouse Parp1^-/-^	Brown adipose tissue (BAT)	Increased NAD^+^ in BAT	*Ucp1* *Ucp3* *Dio2* *Acadm* *Ndufa2* *Ndufb3* *Ndufb5* *Cycs* *Cox17*	uncoupling protein 1 (mitochondrial, proton carrier) uncoupling protein 3 (mitochondrial, proton carrier) deiodinase, iodothyronine, type II acyl-Coenzyme A dehydrogenase, medium chain NADH:ubiquinone oxidoreductase subunit A2 NADH:ubiquinone oxidoreductase subunit B3 NADH:ubiquinone oxidoreductase subunit B5 cytochrome c, somatic cytochrome c oxidase assembly protein 17, copper chaperone
9	DOI: 10.18632/oncotarget.19948 Maternal high calorie diet induces mitochondrial dysfunction and senescence phenotype in subcutaneous fat of newborn mice (42)	Mouse Maternal high calorie diet (8 weeks prior, and during gestation and lactation)	Subcutaneous WAT (SWAT) of newborn mice, at the end of lactation	Decreased NAD^+^/NADH ratio in SWAT of newborn mice, at the end of lactation	*Tnf* *Il6* *Il10* *Adipoq* *mt-Nd6* *mt-Nd4* *mt-Co1*	tumor necrosis factor interleukin 6 interleukin 10 adiponectin, C1Q and collagen domain containing NADH dehydrogenase 6, mitochondrial NADH dehydrogenase 4, mitochondrial cytochrome c oxidase I, mitochondrial
		Mouse Maternal high calorie diet supplemented with niacin (8 weeks prior, and during gestation and lactation)	Subcutaneous WAT (SWAT) of newborn mice, at the end of lactation	Increased NAD^+^/NADH ratio in SWAT of newborn mice, at the end of lactation	*Sod2* *Ucp1* *Tnf* *Il10*	superoxide dismutase 2, mitochondrial uncoupling protein 1 (mitochondrial, proton carrier) tumor necrosis factor interleukin 10
10	DOI: 10.1016/j.bbalip.2020.158819 CD38 downregulation modulates NAD^+^ and NADP(H) levels in thermogenic adipose tissues (53)	Mouse Cold exposure, wild type	Interscapular brown adipose tissue (iBAT)	Increased NAD^+^ in iBAT	*Ppargc1a* *Ucp1* *Nampt* *Cd38* miR-140-3p	peroxisome proliferative activated receptor, gamma, coactivator 1 alpha uncoupling protein 1 (mitochondrial, proton carrier) nicotinamide phosphoribosyltransferase CD38 antigen
		Mouse Cold exposure, Cd38^-/-^	Interscapular brown adipose tissue (iBAT)	Increased NAD^+^ in iBAT	*Ppargc1a* *Ucp1*	peroxisome proliferative activated receptor, gamma, coactivator 1 alpha uncoupling protein 1 (mitochondrial, proton carrier)
11	DOI: 10.1002/mnfr.202100111 Nicotinamide protects against diet-induced body weight gain, increases energy expenditure, and induces white adipose tissue beiging (37)	Mouse High-fat diet supplemented with nicotinamide (NAM)	Inguinal white adipose tissue (iWAT)	Increased NAD^+^ in iWAT Increased NAD^+^/NADH ratio in iWAT	*Adipoq* *Prdm16* *Ucp1* *Ppargc1a* *Ppargc1b* *Mfn2* *Plin1* *Sirt1* *Nmnat1* *Nnmt*	adiponectin, C1Q and collagen domain containing PR domain containing 16 uncoupling protein 1 (mitochondrial, proton carrier) peroxisome proliferative activated receptor, gamma, coactivator 1 alpha peroxisome proliferative activated receptor, gamma, coactivator 1 beta mitofusin 2 perilipin 1 sirtuin 1 nicotinamide nucleotide adenylyltransferase 1 nicotinamide N-methyltransferase
12	DOI: 10.1016/j.jnutbio.2022.109056 Nicotinamide reprograms adipose cellular metabolism and increases mitochondrial biogenesis to ameliorate obesity (63)	Mouse High-fat diet supplemented with nicotinamide (NAM)	Subcutaneous adipose tissue (SCAT)	Increased NAD^+^ in SCAT	*Cox4i1* *Cox8b* *Sirt3* *Sod1* *Sod2* *Acadm* *Acadl* *Ppara* *Ppargc1a* *Tmlhe* *Aldh9a1* *Slc22a5*	cytochrome c oxidase subunit 4I1 cytochrome c oxidase subunit 8B sirtuin 3 superoxide dismutase 1, soluble superoxide dismutase 2, mitochondrial acyl-Coenzyme A dehydrogenase, medium chain acyl-Coenzyme A dehydrogenase, long-chain peroxisome proliferator activated receptor alpha peroxisome proliferative activated receptor, gamma, coactivator 1 alpha trimethyllysine hydroxylase, epsilon aldehyde dehydrogenase 9, subfamily A1 solute carrier family 22 (organic cation transporter), member 5
13	DOI: 10.3389/fendo.2023.1099134 Nicotinamide mononucleotide attenuates HIF-1α activation and fibrosis in hypoxic adipose tissue *via* NAD^+^/SIRT1 axis (28)	Mouse Hypoxia and nicotinamide mononucleotide (NMN) supplementation	Epididymal white adipose tissue (eWAT)	Replenished NAD^+^ levels in eWAT Replenished NAD^+^/NADH ratio in eWAT	*Col1a1* *Col3a1* *Mmp2* *Mmp9* *Timp1* *Lox* *Fn1* *Adipoq* *Lep* *Agt* *Retn* *Il6* *Tgfb1* *Adgre1* *Tnf* *Nos2* *Chil3* *Arg1* *Hif1a^#^*	collagen, type I, alpha 1 collagen, type III, alpha 1 matrix metallopeptidase 2 matrix metallopeptidase 9 tissue inhibitor of metalloproteinase 1 lysyl oxidase fibronectin 1 adiponectin, C1Q and collagen domain containing leptin angiotensinogen resistin interleukin 6 transforming growth factor, beta 1 adhesion G protein-coupled receptor E1 tumor necrosis factor nitric oxide synthase 2, inducible chitinase-like 3 arginase, liver hypoxia inducible factor 1, alpha subunit
14	DOI: 10.1139/cjpp-2022-0454 CD38 deficiency promotes skeletal muscle and brown adipose tissue energy expenditure through activating NAD^+^-Sirt1-PGC1α signaling pathway (30)	Mouse Cd38^-/-^ on high-fat diet	Brown adipose tissue (BAT)	Increased NAD^+^ in BAT	*Sirt1* *Sirt2* *Sirt3* *Ucp1* *Cidea* *Adipoq* *Elovl3* *Ppargc1a* *Dio2* *Tfam* *Nampt*	sirtuin 1 sirtuin 2 sirtuin 3 uncoupling protein 1 (mitochondrial, proton carrier) cell death-inducing DNA fragmentation factor, alpha subunit-like effector A adiponectin, C1Q and collagen domain containing ELOVL fatty acid elongase 3 peroxisome proliferative activated receptor, gamma, coactivator 1 alpha deiodinase, iodothyronine, type II transcription factor A, mitochondrial nicotinamide phosphoribosyltransferase
15	DOI: 10.1007/s13105-021-00851-8 NNMT is induced dynamically during beige adipogenesis in adipose tissues depot-specific manner (64)	Mouse Cold exposure	Interscapular brown adipose tissue (iBAT) Subcutaneous white adipose tissue (sWAT) Epididymal white adipose tissue (eWAT) (Extracted are genes that have significant modulation in any of the studied fat depots.)	Increased NAD^+^ in all studied fat depots	*Nnmt* *Cyp2e1* *Nmnat1* *Nmrk1* *Nampt*	nicotinamide N-methyltransferase cytochrome P450, family 2, subfamily e, polypeptide 1 nicotinamide nucleotide adenylyltransferase 1 nicotinamide riboside kinase 1 nicotinamide phosphoribosyltransferase
16	DOI: 10.1002/iub.2707 Nicotinamide mononucleotide alters body composition and ameliorates metabolic disorders induced by a high-fat diet (44)	Mouse High-fat diet supplemented with nicotinamide mononucleotide (NMN)	Brown adipose tissue (BAT)	Increased NAD^+^ in BAT	*Lipe* *Pparg* *Cebpa* *Ucp1* *Ppargc1a* *Prdm16* *Tnf* *Il6* *Il1b* *Itgam* *Cd68* *Cxcl2* *Adgre1* *Sirt6* *Stk11*	lipase, hormone sensitive peroxisome proliferator activated receptor gamma CCAAT/enhancer binding protein alpha uncoupling protein 1 (mitochondrial, proton carrier) peroxisome proliferative activated receptor, gamma, coactivator 1 alpha PR domain containing 16 tumor necrosis factor interleukin 6 interleukin 1 beta integrin alpha M CD68 antigen C-X-C motif chemokine ligand 2 adhesion G protein-coupled receptor E1 sirtuin 6 serine/threonine kinase 11
17	DOI: 10.1016/j.bbadis.2024.167488 Dietary apigenin ameliorates obesity-related hypertension through TRPV4-dependent vasorelaxation and TRPV4 independent adiponectin secretion (65)	Mouse High-fat diet supplemented with apigenin	Brown adipose tissue (BAT)	Increased NAD^+^ in BAT	*Lamp2* *Ptprc* *Cd38*	lysosomal-associated membrane protein 2 protein tyrosine phosphatase receptor type C CD38 antigen

^#^ Genes that were included for extraction and subsequent bioinformatic analyses in accordance with the study designs of the respective publications.

**Figure 2:**
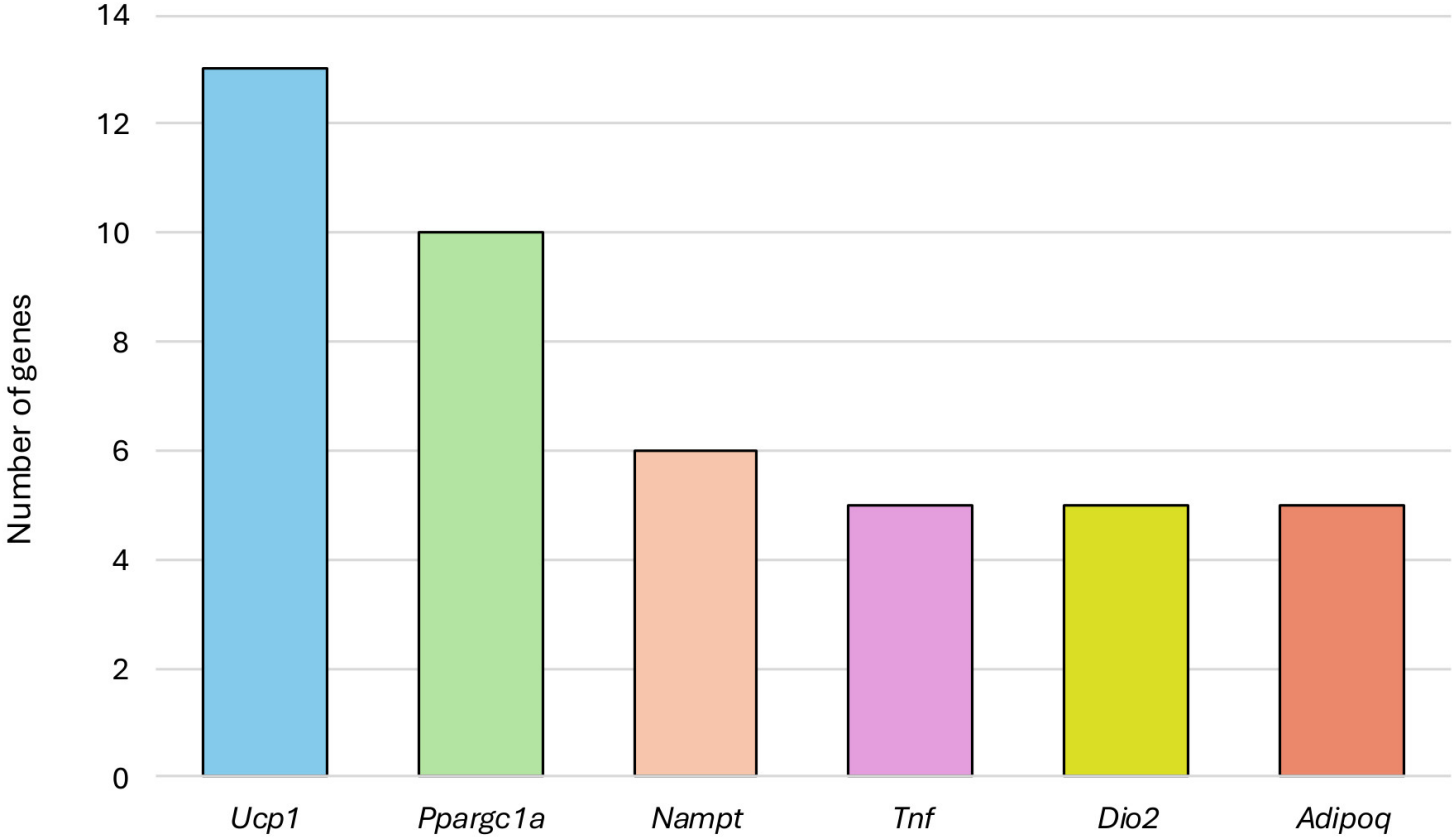
Genes with the highest frequency within the set of differentially expressed genes.

From the eligible studies, a total of 194 differentially expressed protein coding genes and one miRNA were extracted. In addition, two NAD^+^-associated protein coding genes (*Epor* and *Hif1a*) were included for extraction and subsequent functional bioinformatic analyses, in accordance with the study designs of the respective publications (27, 28). The number of unique NAD^+^-associated protein coding genes was 113. The genes with the highest number of occurrences within the set of differentially expressed genes were the following: *Ucp1* (n = 13), *Ppargc1a* (n = 10), *Nampt* (n = 6), *Tnf* (n = 5), *Dio2* (n = 5), and *Adipoq* (n = 5), as shown in **Figure 2**. Overall, these data suggest that studies of NAD^+^ involvement in adipose tissue (dys)function were predominantly focused on its roles in mitochondrial activity and thermogenesis (*Ucp1, Ppargc1a, Dio2)*, inflammation (*Tnf, Adipoq*), or insulin sensitivity (*Adipoq*).

### 3.2 Pathway enrichment analysis

**Figure 3:**
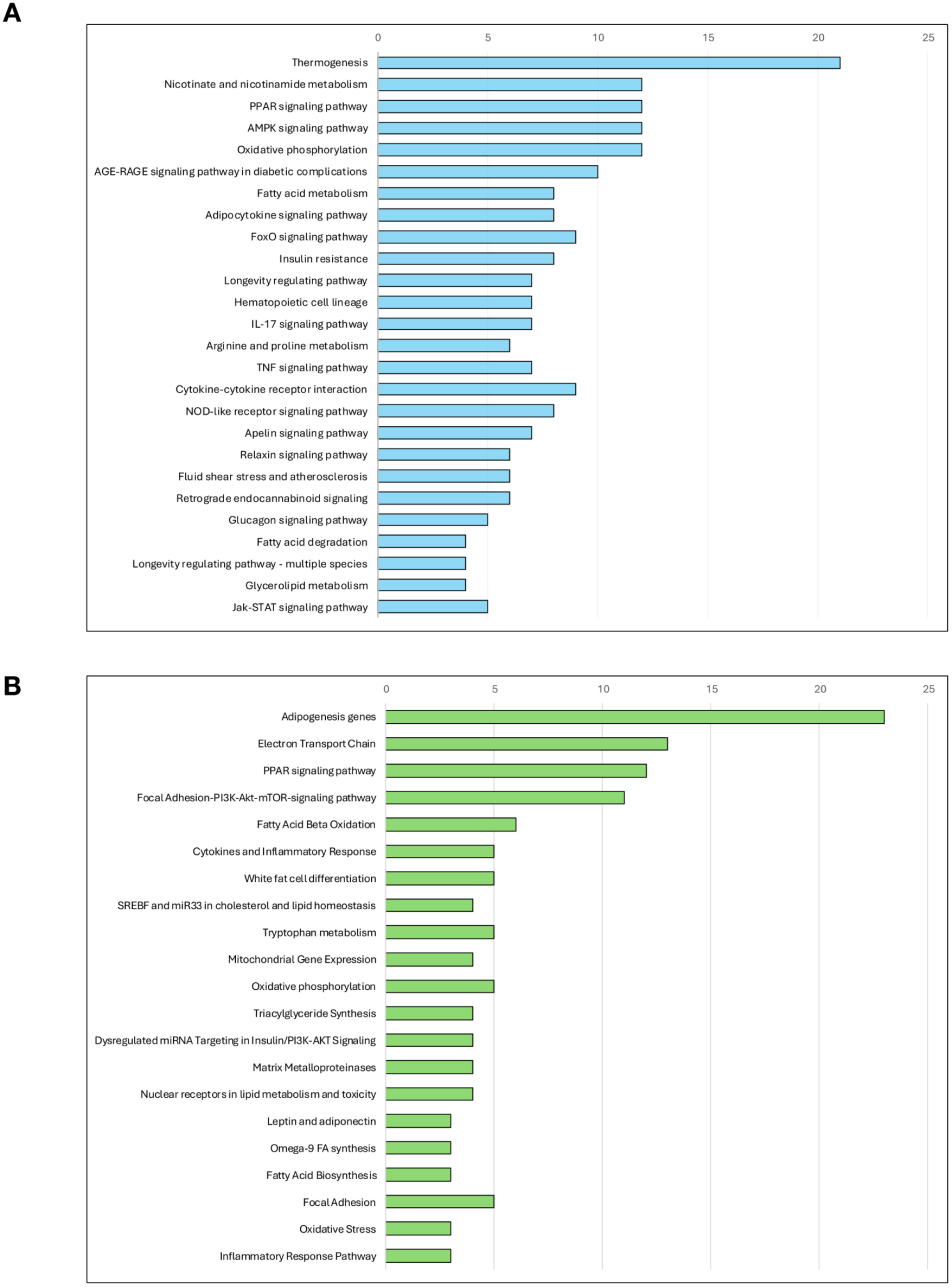
NAD^+^-associated top cellular pathways in rodent adipose tissue, identified in **(A)** KEGG database (n = 26) and **(B)** WikiPathways (n = 21). The analysis was conducted using the bioinformatic tool GeneTrail3.2. Pathways are arranged according to the adjusted p-value (with the lowest p-value at the top); the x-axis represents the number of genes associated with each pathway.

To gain insight into biological functions of the extracted protein coding genes, a GeneTrail pathway enrichment analysis was performed, revealing a substantial number of canonical pathways significantly associated with our gene set. After excluding pathways related to chronic diseases, infectious diseases, and cancer, we focused on the highest-ranked cellular pathways identified in each of the two databases. Interrogation of the KEGG database identified the following pathways with the highest number of hits: *thermogenesis* (n = 21 hits), *nicotinate and nicotinamide metabolism* (n = 12 hits), *PPAR signaling pathway* (n = 12 hits), *AMPK signaling pathway* (n = 12 hits), *oxidative phosphorylation* (n = 12 hits), and *AGE-RAGE signaling pathway in diabetic complications* (n = 10 hits). Interrogation of the WikiPathways database revealed the following top cellular pathways: *adipogenesis genes* (n = 23 hits), *electron transport chain* (n = 13 hits), *PPAR signaling pathway* (n = 12 hits), and *focal adhesion-PI3K-Akt-mTOR signaling pathway* (n = 11 hits). Enriched KEGG pathways also included the *IL-17 signaling pathway*, the *TNF signaling pathway*, *cytokine-cytokine receptor interaction*, and the *NOD-like receptor signaling pathway*, while enriched WikiPathways included the *cytokines and inflammatory response* and the *inflammatory response pathway*. **Figures 3A, B** present the top 26 and 21 cellular pathways associated with the extracted gene set in KEGG and WikiPathways, respectively, ranked according to statistical significance, with the number of genes associated with each pathway shown on the x-axis. Additionally, the genes (hits) that were mapped to each of these pathways are listed in **Supplementary Table 1**.

To corroborate the results of the GeneTrail pathway enrichment analysis, the same gene set was re-analyzed using the bioinformatic tool Enrichr for interrogation of the *WikiPathways 2024 Mouse* database. This analysis yielded nearly identical results, which are presented in **Supplementary Table 2**. The table compares the top 30 enriched WikiPathways identified by both GeneTrail and Enrichr. Of these, 24 pathways are shared between the two analyses (presented in blue in the table) with similar levels of statistical significance, indicating strong concordance. Two additional pathways show a high degree of similarity (presented in orange), while only four pathways are unique to one database within the top 30. Overall, this high degree of overlap supports the robustness of GeneTrail pathway enrichment analysis.

**Figure 4:**
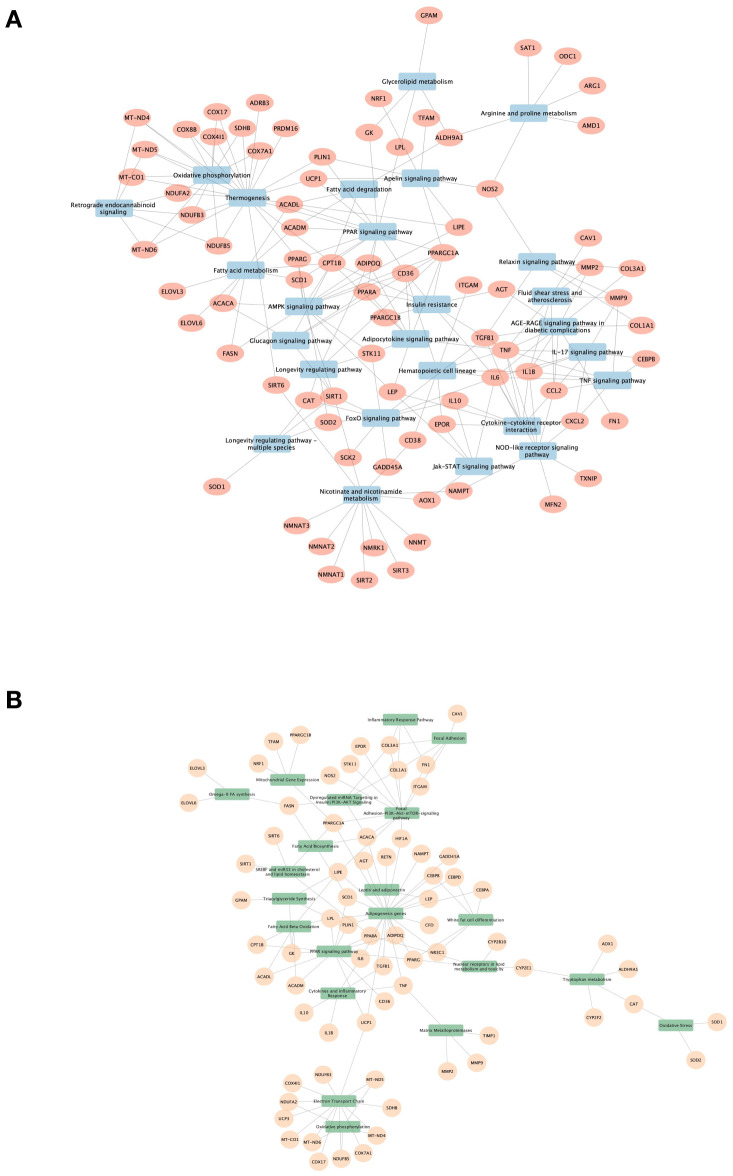
Gene-pathway networks of NAD^+^-associated top cellular pathways in rodent adipose tissue, identified in **(A)** KEGG database and **(B)** WikiPathways. Genes associated with top canonical pathways were identified using the bioinformatic tool GeneTrail3.2. Networks of gene-pathway interactions were built using the Cytoscape software, version 3.10.4.

Furthermore, to identify interactions among the highest-ranked cellular pathways, we built and visualized gene-pathway networks for the top pathways from each of the two databases, KEGG and WikiPathways, as shown in **Figures 4A, B**, respectively. Interestingly, among KEGG pathways, the *nicotinate and nicotinamide metabolism pathway* appears only marginally in the network but has the potential to affect the broader network of metabolic and vascular-related pathways through the involvement of Cd38 and sirtuins. On the other hand, among WikiPathways, the *adipogenesis genes* is the pathway that occupies a central position in the network and exhibits the highest number of hits.

### 3.3 Protein-protein interactions

Our next step was to perform protein-protein interaction analysis with the aim to identify genes with highest potential biological impact. This analysis identified the proteins with the highest number of interactions within the network, which include: Pparg (n = 32), Ppargc1a (n = 32), Tnf (n = 30), Il1b (n = 28), Il6 (n = 28), Adipoq (n = 27), Ppara (n = 26), Lep (n = 25), Sirt1 (n = 24), Ccl2 (n = 21), Il10 (n = 21), Cd36 (n = 18), Tgfb1 (n = 18), Hif1a (n = 16), Lipe (n = 16), Itgam (n = 16), Cidea (n = 16), Ucp1 (n = 15), Mmp9 (n = 15), and Cox4i1 (n = 15). The remaining proteins exhibited fewer than 15 interactions, while 12 proteins showed no interactions with other proteins.

**Figure 5:**
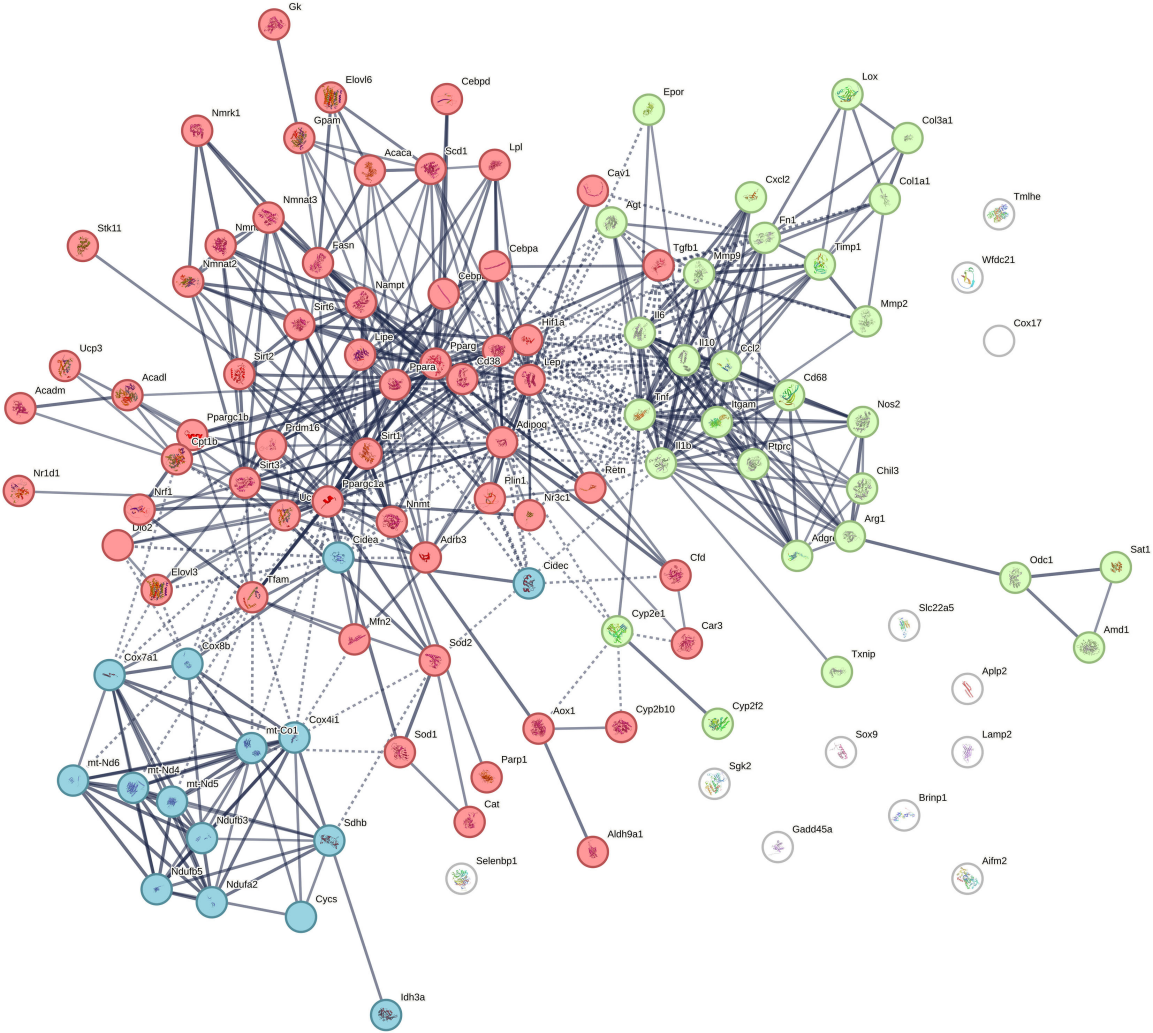
Clusters of protein-protein interactions. Analysis was conducted using the STRING database. Proteins are organized in 3 functional groups: cluster 1 (red), associated with the positive regulation of cold-induced thermogenesis; cluster 2 (green), associated with inflammation; and cluster 3 (blue), associated with cellular respiration.

Using the functionality of the STRING database to organize proteins into functional groups, three distinct clusters were identified: Cluster 1 (red), associated with the positive regulation of cold-induced thermogenesis; Cluster 2 (green), related to inflammation; and Cluster 3 (blue), encompassing proteins involved in cellular respiration (**Figure 5**). Cluster 1 contained the largest number of proteins (n = 58), followed by Cluster 2 (n = 28) and Cluster 3, which included the fewest proteins (n = 15). Within Cluster 1, the proteins with the highest number of interactions were Pparg (n = 32) and Ppargc1a (n = 32), in Cluster 2, it was Tnf (n = 30), and in Cluster 3, Cidea showed the highest number of interactions (n = 16). A detailed presentation of the protein-protein interactions is provided in **Supplementary Table 3**.

### 3.4 Identification of master regulators and genes associated with human diseases

Our next objective was to identify the master regulators of the extracted NAD^+^-associated protein coding genes by interrogating the Qiagen IPA database. The analysis revealed a diverse array of regulators, such as transcription factors, transporters, growth factors, receptors, enzymes, microRNAs, and a broad range of endogenous and exogenous chemical compounds, all showing strong regulatory potential over NAD^+^-associated protein coding genes in rodent adipose tissue. The most significant regulators, characterized by the lowest p-values and the highest number of associated genes (hits), included PRDM16 (62 hits), CAB39L (60 hits), rosiglitazone (54 hits), the MEF2C,D:PPARGC1A complex (52 hits), or LEP (47 hits), as well as several key metabolic compounds (AMP, D-glucose, or 8,9-epoxyeicosatrienoic acid). The complete list of the top 50 master regulators is presented in **Table 2**.

**Table 2:** Top master regulators of NAD^+^-associated protein coding genes in the adipose tissue.

Molecule type	Master regulator	p-value	Number of hits
*Transcription regulator*	PRDM16	1.37E-55	62
	NRIP1	9.09E-53	55
	PPARGC1A	2.58E-51	46
	ZNF423	1.86E-48	43
	SMARCD3	6.61E-47	48
	ASXL1	1.03E-45	41
*Complex*	MEF2C,D:PPARGC1A	1.63E-55	52
*Group*	PPARGC1A (family)	6.31E-53	47
	SMAD1/5/9 (family)	1.26E-51	47
	RELAXIN (family)	8.05E-50	50
*Transporter*	ABCC1	5.50E-47	45
	SLC5A5	1.85E-46	49
	FABP1	8.41E-46	51
*Growth factor*	LEP	1.03E-55	47
	BMP10	1.29E-53	50
*G-protein coupled receptor*	OXGR1	1.18E-46	49
*Kinase*	CAB39L	1.33E-59	60
	SPEG	7.70E-53	47
*Enzyme*	DUT	1.16E-47	50
*microRNA*	mir-103 (includes others)	3.10E-51	55
*Chemical - endogenous mammalian*	AMP	4.14E-50	44
	D-glucose	2.34E-49	50
	8,9-epoxyeicosatrienoic acid	3.81E-49	50
	acetyl-coenzyme A	6.81E-47	38
	D-fructose	1.63E-46	49
	13-hydroxyoctadecadienoic acid	5.10E-46	49
*Chemical - endogenous non-mammalian*	isohumulone	1.16E-47	50
*Chemical drug*	rosiglitazone	2.24E-66	54
	farglitazar	5.89E-49	51
	vismodegib	1.56E-48	48
	reglitazar	1.16E-47	50
	tesaglitazar	1.16E-47	50
	aleglitazar	1.16E-47	50
	vipoglanstat	1.27E-46	44
	ezetimibe	3.07E-46	41
*Chemical reagent*	DMH1	1.19E-53	49
	10,12-tricosadiynoic acid	1.36E-47	46
	BMS-309403	4.24E-47	43
	4-hydroxymethyl-piperidine-1-carboxylic acid 4-(5-trifluoromethylpyridin-2-yloxy)-phenyl ester	1.10E-46	49
	WWL11	1.18E-46	49
	12-(3-adamantan-1-yl-ureido) dodecanoic acid	1.20E-46	50
	eicosanoid	4.49E-46	49
	high erucic acid rapeseed oil	1.07E-45	42
*Chemical toxicant*	mono-(2-ethylhexyl)phthalate	4.64E-49	59
*Other*	C2CD5	2.25E-52	53
	CIDEC	3.23E-52	48
	PLIN5	6.34E-47	44
	PLIN1	1.52E-46	49
	LANCL2	5.24E-46	47
	LINC00963	9.68E-46	48

We further performed a disease-association analysis of the extracted NAD^+^-related protein coding genes (**Table 3**). The results demonstrated strong enrichment for nutritional and metabolic diseases, showing the highest significance (58 hits, p = 1.47E-50). Substantial associations were also observed across multiple metabolic disorder categories, including metabolic diseases, glucose metabolism disorders, nutrition disorders, obesity, overweight, and overnutrition. In addition, the genes were robustly linked to a range of cardiovascular conditions, such as heart diseases (52 hits), cardiovascular diseases (54 hits), vascular diseases (42 hits), and hypertension (28 hits). Strong associations were also detected with digestive system diseases, particularly liver diseases (53 hits), as well as with endocrine system diseases, including diabetes mellitus (32 hits). Together, these results indicate that NAD^+^-associated protein coding genes are involved in a broad spectrum of pathophysiological processes relevant to metabolism, cardiovascular regulation, and systemic human diseases.

**Table 3:** Association of extracted protein coding genes with human diseases.

Disease name	Disease ID	Disease categories	Corrected p-value	Number of hits
Nutritional and Metabolic Diseases	MESH:D009750	Nutritional and Metabolic Diseases	1.47E-50	58
Heart Diseases	MESH:D006331	Cardiovascular disease	6.33E-49	52
Cardiovascular Diseases	MESH:D002318	Cardiovascular disease	3.70E-45	54
Digestive System Diseases	MESH:D004066	Digestive system disease	2.98E-43	63
Nutrition Disorders	MESH:D009748	Nutrition disorder	5.34E-42	32
Metabolic Diseases	MESH:D008659	Metabolic disease	1.14E-40	50
Body Weight	MESH:D001835	Signs and symptoms	1.38E-39	33
Obesity	MESH:D009765	Nutrition disorder | Signs and symptoms	2.19E-39	29
Overnutrition	MESH:D044343	Nutrition disorder	2.19E-39	29
Overweight	MESH:D050177	Nutrition disorder | Signs and symptoms	2.19E-39	29
Liver Diseases	MESH:D008107	Digestive system disease	8.47E-39	53
Vascular Diseases	MESH:D014652	Cardiovascular disease	5.09E-38	42
Endocrine System Diseases	MESH:D004700	Endocrine system disease	3.77E-37	44
Diabetes Mellitus	MESH:D003920	Endocrine system disease | Metabolic disease	5.18E-36	32
Hypertension	MESH:D006973	Cardiovascular disease	5.58E-36	28
Fibrosis	MESH:D005355	Pathology (process)	1.49E-35	41
Glucose Metabolism Disorders	MESH:D044882	Metabolic disease	2.21E-35	32

## 4 Discussion

**Figure 6:**
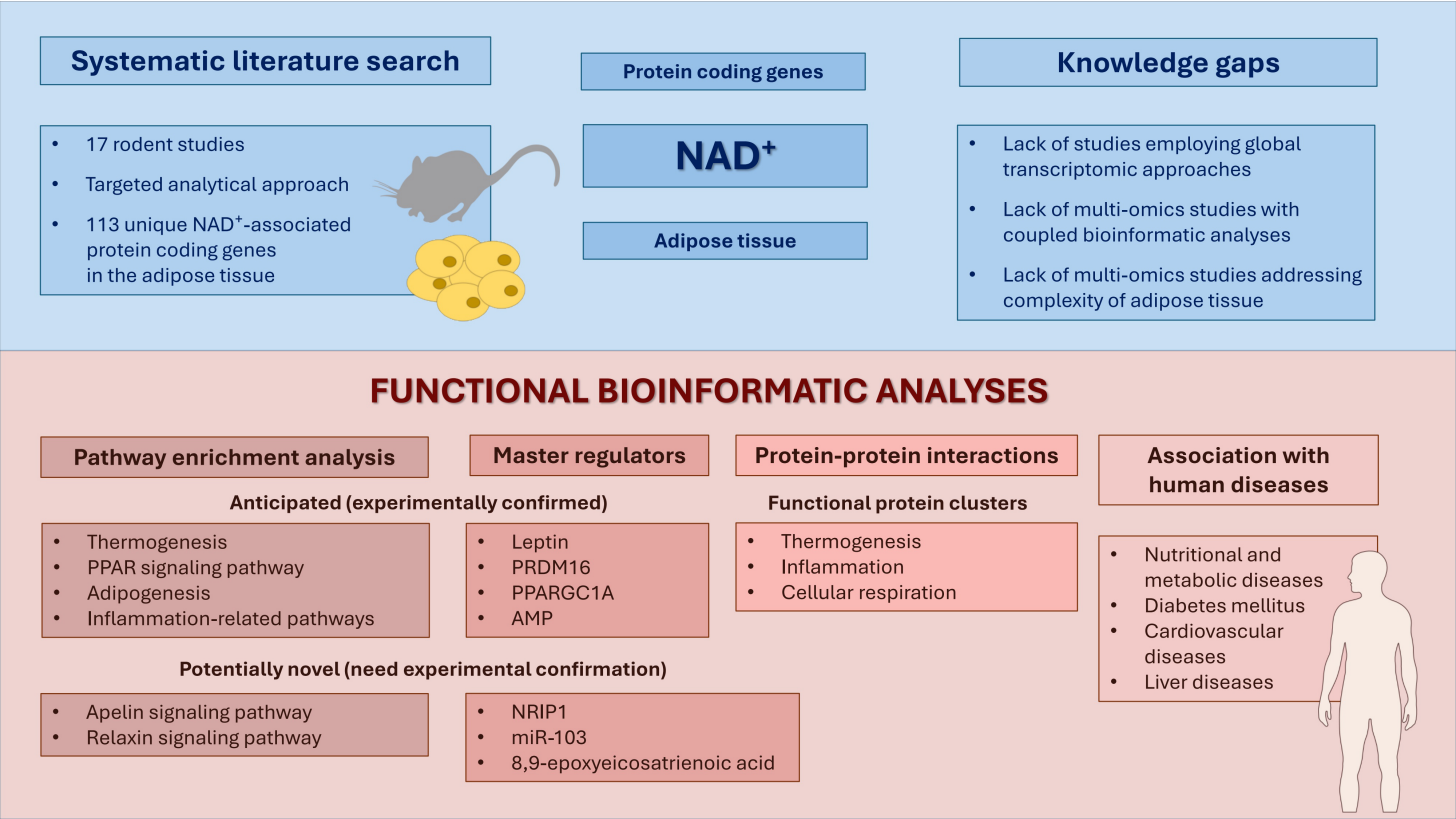
Summary figure. Identification of both established and potentially novel molecular mechanisms, knowledge gaps, and directions for further research through a systematic literature search and integrative functional bioinformatic analysis of NAD^+^-associated protein coding genes in rodent adipose tissue (66, 67).

Given the central role of adipose tissue in the regulation of whole-body metabolism and insulin sensitivity, elucidating the molecular mechanisms of its (dys)function has become an important area of investigation. In this study, we aimed to collect and synthesize available evidence on the role of NAD^+^ in adipose biology by conducting a systematic review of experimental rodent studies, followed by integrative functional analyses of extracted NAD^+^-associated protein coding genes using multiple bioinformatic approaches. The summary of our findings is presented in **Figure 6**.

Our pathway enrichment analysis identified *thermogenesis* as the most significantly enriched KEGG pathway associated with extracted NAD^+^-related protein coding genes. Notably, brown and beige adipocytes are central mediators of non-shivering thermogenesis, primarily in response to cold exposure and, to a lesser extent, to other stimuli such as meal intake or thermogenic food compounds (29). Multiple studies identified in our systematic literature search report a positive association between intracellular NAD^+^ levels and *Ucp1* gene expression, highlighting the critical role of NAD^+^ in the regulation of non-shivering thermogenesis. This process is mediated, at least in part, by SIRT1, which enhances PGC1α-driven transcriptional programs that promote UCP1 induction and mitochondrial biogenesis (PGC1α – PPARG Coactivator 1 Alpha; official gene symbol is PPARGC1A) (30). Beyond its role in cold adaptation, thermogenic adipose tissue has emerged as a dynamic “metabolic sink” for excess nutrients, thereby helping to protect against weight gain and metabolic dysfunction (31). Accordingly, activation of BAT and induction of WAT beiging are considered promising strategies to reduce adiposity and mitigate metabolic syndrome and type 2 diabetes mellitus (32). Upon activation, BAT also contributes to lowering plasma triglyceride levels through lipoprotein lipase-mediated hydrolysis of circulating triglycerides, followed by uptake of the liberated fatty acids *via* transporters such as CD36 (10). Collectively, these mechanisms highlight the pivotal role of thermogenic adipose tissue in maintaining systemic metabolic homeostasis and point to NAD^+^ as an important contributor to these processes.

The *peroxisome proliferator-activated receptor (PPAR) signaling pathway* is also among the top pathways associated with our set of NAD^+^-related protein coding genes and is enriched with high statistical significance in both interrogated databases. In addition, in our protein-protein interaction analysis, Pparg, together with its coactivator Ppargc1a, emerged as the node with the highest number of interactions within the overall STRING network, as well as within the cluster associated with the positive regulation of cold-induced thermogenesis. PPARγ (PPARG) is expressed in both white and brown adipose tissues and plays a central role in adipocyte differentiation and functional regulation (33). In brown adipose tissue, PPARγ promotes thermogenesis and mitochondrial biogenesis primarily *via* its interaction with PRDM16 and PGC1α (34, 35). In white adipose tissue, PPARγ contributes to its beiging, which occurs *via* SIRT1-mediated deacetylation of PPARγ at Lys268 and Lys293. This post-translational modification leads to recruitment of PRDM16, which, in cooperation with PPARγ, induces a BAT-specific gene expression signature while repressing WAT-specific genes (36). Consistent with this mechanism, a study identified in our systematic literature search reported a positive correlation between intracellular NAD^+^ levels and the expression of *Sirt1, Prdm16*, *Ucp1*, *Ppargc1a*, and *Ppargc1b* in beiging inguinal white adipose tissue (37). Overall, the regulatory role of SIRT1-mediated deacetylation of PPARγ highlights the importance of maintaining adequate NAD^+^ levels in adipose tissue to support thermogenic remodeling and metabolic function.

The *adipogenesis genes* is the cellular pathway most significantly associated with our gene set in the WikiPathways database and occupies a central position within the corresponding gene-pathway network. Biologically, adipogenesis is governed by a tightly regulated transcriptional cascade in which PPARγ and CCAAT/enhancer binding proteins (C/EBP) play key roles. Activation of this adipogenic gene network is essential for the maintenance of metabolic homeostasis, as it enables WAT to expand through the formation of new, functional adipocytes in response to caloric excess (38–40). In metabolically healthy individuals, WAT expansion is characterized predominantly by adipocyte hyperplasia, *i.e.*, the generation of numerous small, insulin-sensitive adipocytes accompanied by adequate vascularization. This adaptive remodeling preserves lipid storage capacity and prevents accumulation of fat in non-adipose tissues. In contrast, impaired adipogenesis limits the capacity of WAT depots to expand appropriately, resulting in adipocyte hypertrophy, local hypoxia, extracellular matrix remodeling and fibrosis, and increased infiltration of pro-inflammatory cells. These pathological changes are associated with reduced adiponectin levels and enhanced ectopic lipid deposition in the liver and skeletal muscle, thereby promoting insulin resistance and metabolic dysfunction (41).

Pathway enrichment analysis identified multiple inflammatory pathways significantly associated with our set of NAD^+^-related protein coding genes across both examined databases. Consistent with these findings, our protein-protein interaction analysis revealed a robust cluster of inflammatory proteins, with Tnf, Il1b, and Il6 occupying central positions, exhibiting the highest number of interactions with other proteins within the network. Collectively, the results of these analyses support an association between intracellular NAD^+^ levels and adipose tissue inflammation. More specifically, the association between NAD^+^ and the degree of adipose tissue inflammation has been examined in four publications (28, 42–44) that were included in our functional bioinformatic analysis. Across all of these studies, an inverse relationship was consistently observed between intracellular NAD^+^ levels and the expression of inflammatory genes, particularly, *Tnf*, *Il1b*, and *Il6*, indicating the critical role of NAD^+^ in adipose biology. Inflammation is one of the key hallmarks of adipose tissue dysfunction, not only in obesity, where adipose tissue dysfunction has been most extensively studied, but also during ageing (45). Notably, aged adipose tissue exhibits a pro-inflammatory dysfunctional profile similar to that observed in obesity. In ageing, however, inflammation is a hallmark of a distinct senescent adipose tissue phenotype, which also drives systemic consequences, including insulin resistance and cardiometabolic disorders (45).

Beyond the canonical pathways whose significant association with NAD^+^-related protein coding genes in adipose tissue was, to some extent, expected, given the biological processes examined in the studies included in our functional bioinformatic analysis, we also identified several cellular pathways that have not previously been investigated in the context of NAD^+^ function in adipose tissue, including the *apelin signaling pathway* and the *relaxin signaling pathway*. Notably, none of the studies included in our functional bioinformatic analysis explicitly mentioned apelin, relaxin, or their respective signaling pathways. Regarding apelin and its signaling pathway, a substantial body of literature supports their relevance not only in adipose biology but also in systemic metabolic regulation (46). In adipose biology, apelin has been shown to promote brown adipocyte differentiation, alleviate TNFα-mediated inhibition of brown adipogenesis, increase the basal activity of brown adipocytes, and induce brown-like characteristics in white adipocytes (47). Moreover, apelin suppresses the production and release of reactive oxygen species (ROS) and mitigates oxidative stress-induced cellular dysfunction in adipocytes (48). In addition, apelin contributes to the development of a functional vascular network within adipose tissue, a process stimulated by hypoxic conditions (49). Whether the *apelin signaling pathway* is directly or indirectly linked to intracellular NAD^+^ concentrations in adipose tissue remains unknown and warrants investigation in future experimental studies. In contrast to apelin, available data on the role of relaxin in adipose tissue are extremely limited. To date, a study by Yamamoto et al. demonstrated that relaxin is secreted by cells of the stromal vascular fraction of adipose tissue and plays a role in regulating lipid accumulation in adipocytes (50). Particularly relevant preclinical evidence is provided by the study of Aragón-Herrera et al., who administered human recombinant relaxin-2 (serelaxin) to rats. They demonstrated that serelaxin, a novel drug with pleiotropic cardiovascular effects, can regulate visceral adipose tissue (VAT) metabolism. Specifically, the study showed that a two-week serelaxin treatment affected VAT lipolysis by increasing mRNA expression of hormone-sensitive lipase while decreasing expression of adipose triglyceride lipase, accompanied by reduced VAT expression of the fatty acid transporter CD36. Serelaxin also exerted an anti-inflammatory effect in VAT, as evidenced by decreased mRNA expression of TNFα, IL-1β, chemerin, and the chemerin receptor (51). Interestingly, in our study relaxin also emerged as one of the master regulators of the extracted gene set. However, as with apelin, future studies are required to determine whether relaxin is directly or indirectly associated with intracellular NAD^+^ levels in adipose tissue.

Similar to the pathway enrichment analysis, the master regulator analysis of NAD^+^-associated protein coding genes identified several master regulators whose involvement was anticipated, including leptin, the transcription factors PRDM16 and PPARGC1A, metabolites such as AMP, glucose, and fructose, and the antidiabetic drug rosiglitazone. However, this analysis also pinpointed several new potential master regulators whose direct functional roles in the context of NAD^+^ involvement in adipose biology have not been investigated. These include nuclear receptor interacting protein 1 (NRIP1), zinc-finger protein 423 (ZNF423), miR-103, 8,9-epoxyeicosatrienoic acid, and 13-hydroxyoctadecadienoic acid. For some candidates, such as calcium binding protein 39-like (CAB39L), no studies directly linking them to adipose tissue biology are currently available. However, it is interesting to note that CAB39L functions as a metabolic checkpoint regulating cellular metabolism and has been linked to AMPK activation. Although the direct evidence connecting CAB39L to metabolism originates from studies on tumor suppression, the pathway it regulates, LKB1-AMPK-PGC1α, may be implicated in the role of NAD^+^ in adipose biology (52). Nevertheless, this hypothesis requires validation through targeted experimental studies.

Notably, in addition to screening the eligible studies for extraction of NAD^+^-associated protein coding genes, we also considered miRNAs and other non-coding RNAs for data extraction. However, only a single miRNA, miR-140-3p, was identified as NAD^+^-associated, which was insufficient for inclusion in subsequent functional bioinformatic analyses. Nevertheless, this observation highlights a substantial knowledge gap in current research investigating the biological role of NAD^+^ in adipose tissue, which should be addressed in future studies. A study included in our functional bioinformatic analysis demonstrated that miR-140-3p is upregulated in interscapular brown adipose tissue of wild-type mice following cold exposure, concomitant with reduced expression of Cd38 and increased NAD^+^ levels (53). To the best of our knowledge, this represents the only study directly reporting a metabolic role for miR-140-3p in adipose tissue. However, complementary evidence supports the functional link between miR-140-3p and CD38 regulation. Specifically, miR-140-3p has been shown to downregulate TNFα-induced CD38 overexpression in human airway smooth muscle cells (54). In addition, it has been demonstrated that miR-140-3p suppresses macrophage proliferation and migration (55), a finding of particular relevance for adipose biology, given the central role of immune cell-adipocyte interactions in adipose tissue and metabolic regulation.

Our systematic literature search revealed that, among studies investigating the molecular mechanisms underlying the involvement of NAD^+^ in adipose tissue (dys)function, studies employing global transcriptomic approaches remain scarce, which substantially limits our understanding of the role of NAD^+^ in adipose biology. Given the pivotal role of NAD^+^ in adipose tissue (dys)function, future research is expected to place considerably greater emphasis on analyzing global transcriptomic modulations, which have the potential to uncover novel and previously unrecognized molecular mechanisms. Nevertheless, targeted gene expression analyses remain a powerful and complementary analytical approach for validating and further refining findings derived from global transcriptomic studies. In addition, global transcriptomic analyses offer unique advantages, as they provide insight not only into protein coding genes but also into diverse populations of non-coding RNAs, which are also likely to be modulated in biological processes associated with changes in NAD^+^ level in adipose tissue. Moreover, considering the complex and diverse cellular composition of adipose tissue, future studies would substantially benefit from the application of single-cell transcriptomics as a powerful analytical approach to elucidate global transcriptomic modulations across distinct adipose tissue cell populations. Parallel analyses of global transcriptomic, proteomic, and metabolomic modulations, through the implementation of integrated multi-omics approaches coupled with advanced bioinformatic methods, will enable a more comprehensive understanding of the biological roles of NAD^+^ in adipose tissue. Ultimately, applying these advanced analytical and bioinformatic approaches to specific adipose tissue phenotypes and different adipose depots will create a detailed and comprehensive map of NAD^+^ involvement in the adipose tissue (dys)function, and therefore overcome the main limitation of our study, which is a general analysis of NAD^+^-associated protein coding genes in the adipose tissue. Notably, with our general analysis, some identified pathways or master regulators could have a stronger influence on the metabolism of one type of adipose tissue than in others.

In conclusion, our meta-analysis of extracted NAD^+^-related protein coding genes identified several well-established canonical pathways and master regulators associated with NAD^+^ involvement in adipose tissue (dys)function. Notably, we also identified less anticipated pathways, such as the *apelin signaling pathway* and the *relaxin signaling pathway*. The potential involvement of these pathways in NAD^+^-associated adipose tissue biology warrants further detailed investigation. An in-depth and comprehensive understanding of the biological role of NAD^+^ in adipose tissue is of particular importance for identifying potential molecular targets for the development of effective and safe NAD^+^-boosting strategies. Such approaches may mitigate the detrimental metabolic consequences of obesity and may also hold promise for promoting healthy ageing and longevity.

## Data Availability

The original contributions presented in the study are included in the article/**Supplementary Material**. Further inquiries can be directed to the corresponding author.
